# Intraventricular or intrathecal polymyxin B for treatment of post-neurosurgical intracranial infection caused by carbapenem-resistant gram-negative bacteria: a 8-year retrospective study

**DOI:** 10.1007/s10096-024-04794-y

**Published:** 2024-03-05

**Authors:** Yangmin Hu, Danyang Li, Gensheng Zhang, Yunjian Dai, Meng Chen, Huifang Jiang, Wei Cui

**Affiliations:** 1 Department of Pharmacy, Second Affiliated Hospital, Zhejiang University School of Medicine, Hangzhou, 310003 China; 2 Department of Critical Care Medicine, Second Affiliated Hospital, Zhejiang University School of Medicine, Hangzhou, 310003 China

**Keywords:** Central nervous system infection, Intraventricular, Polymyxin B, Gram-negative bacteria, Multidrug resistance

## Abstract

**Purpose:**

Post-neurosurgical intracranial infection caused by carbapenem-resistant gram-negative bacteria (CRGNB) is a life-threatening complication. This study aimed to assess the current practices and clinical outcomes of intravenous (IV) combined with intraventricular (IVT)/intrathecal (ITH) polymyxin B in treating CRGNB intracranial infection.

**Methods:**

A retrospective study was conducted on patients with post-neurosurgical intracranial infection due to CRGNB from January 2013 to December 2020. Clinical characteristics and treatment outcomes were collected and described. Kaplan–Meier survival and multivariate logistic regression analyses were performed.

**Results:**

The study included 114 patients, of which 72 received systemic antimicrobial therapy combined with IVT/ITH polymyxin B, and 42 received IV administration alone. Most infections were caused by carbapenem-resistant *Acinetobacter baumannii* (CRAB, 63.2%), followed by carbapenem-resistant *Klebsiella pneumoniae* (CRKP, 31.6%). Compared with the IV group, the IVT/ITH group had a higher cerebrospinal fluid (CSF) sterilization rate in 7 days (*p* < 0.001) and lower 30-day mortality (*p* = 0.032). In the IVT/ITH group, patients with CRKP infection had a higher initial fever (*p* = 0.014), higher incidence of bloodstream infection (*p* = 0.040), lower CSF sterilization in 7 days (*p* < 0.001), and higher 30-day mortality (*p* = 0.005) than those with CRAB infection. Multivariate logistic regression analysis revealed that the duration of IVT/ITH polymyxin B (*p* = 0.021) was independently associated with 30-day mortality.

**Conclusions:**

Intravenous combined with IVT/ITH polymyxin B increased CSF microbiological eradication and improved clinical outcomes. CRKP intracranial infections may lead to more difficult treatment and thus warrant attention and further optimized treatment.

## Introduction

Post-neurosurgical central nervous system (CNS) infection, a serious complication of neurosurgical procedures, is associated with high mortality, morbidity, and poor functional outcomes [1, 2]. In recent years, gram-negative pathogen-related ventriculitis/meningitis have been reported more frequently and have had worse clinical characteristics and prognostic outcomes than gram-positives [3–5]. To make things worse, the incidence of carbapenem-resistant gram-negative bacteria (CRGNB) is increasing worldwide [6, 7]. Clinically related CRGNB mainly include *Acinetobacter baumannii*, *Pseudomonas aeruginosa*, and *Klebsiella pneumoniae*, which are resistant to piperacillin, third-generation cephalosporins, carbapenems, and fluoroquinolones [8]. For the treatment of CRGNB infection, clinicians often resort to combination therapy based on polymyxins (colistin or polymyxin B), aminoglycosides, and tigecycline [9, 10]. However, these antibiotics were found to have inadequate penetration from the blood–brain barrier, making it difficult to achieve adequate cerebrospinal fluid (CSF) concentrations using systemic therapy [11]. Therefore, the choice of antibiotics for CRGNB ventriculitis/meningitis is extremely limited, greatly increasing the difficulty of treatment.

The presence of CRGNB has forced the use of topical therapies to achieve an effective therapeutic concentration of antibiotics at the site of infection [12]. Intraventricular (IVT) and intrathecal (ITH) therapies are widely recognized treatments for ventriculitis and meningitis, especially when the pathogen is carbapenem-resistant bacteria [13]. Such therapies bypass the blood–brain and blood–CSF barriers and overcome the limited penetration of antibiotics into the CSF, raise the respective drug concentrations, and minimize systemic toxicities [11, 14]. The current guideline recommends that the adjunct IVT/ITH antimicrobial therapy should be reserved for patients with infections caused by multidrug-resistant (MDR) gram-negative bacteria or for those who poorly respond to the standard intravenous agents [15]. Several studies have chosen IVT/ITH administration to achieve an adequate level of drugs in the CSF to combat these resistant organisms [16–19].

Because of its potent activity against CRGNB, polymyxin B is used as the last therapy after all other treatment schemes fail [20]. However, most previous studies have focused on the treatment of MDR *A. baumannii* infections with small samples, whereas the treatment of intracranial infections caused by other MDR gram-negative bacteria is extremely limited. In addition, there is a dearth of concrete evidence to determine the optimal dose and duration of IVT/ITH polymyxin B for patients with intracranial infection caused by CRGNB. To further confirm the efficacy and safety of the IVT/ITH strategy, we retrospectively analyzed the clinical features and eventual outcomes of 114 patients with post-neurosurgical intracranial infection caused by CRGNB.

## Methods

### Patients and definition

This retrospective study was conducted from January 2013 to December 2020 at the Second Affiliated Hospital of Zhejiang University, School of Medicine, which is a tertiary care hospital. All adult patients with a positive CSF culture for CRGNB after neurosurgery were enrolled. Patients with any of the following conditions were excluded: (i) less than 18 years of age; (ii) a polymicrobial result from CSF culture; (iii) possible contamination; (iv) IVT/ITH polymyxin B only once after the diagnosis of intracranial infection; or (v) incomplete clinical data. Each patient was included in the study only once at the time of the first positive CSF culture. The diagnosis and treatment management of intracranial infections and evaluation of the final outcomes were determined by physicians. After inclusion, patients were classified into two groups: patients receiving intravenous (IV) plus IVT/ITH polymyxin B (IVT/ITH group) and those who received only IV treatment (IV group). In the IVT/ITH group, the process of local injection of polymyxin B was that the clinician removed 5 mL of CSF via the ventricular drainage tube or lumbar cistern drainage tube and discarded it, then injected 5 mg/day of polymyxin B and finally closed the tube for 2 h. The clinician determined whether or not to change the dosing interval based on CSF results. Approval for this study was granted with waiver of individual consent by the Ethics Committee of the 2nd Affiliated Hospital, School of Medicine, Zhejiang University (No. 2023 − 0193).

CRGNB intracranial infection was defined in such a way that a positive culture of CRGNB from CSF cultures was found at least once, with increased white cells, decreased glucose, and/or increased protein levels in the CSF, as well as clinical symptoms suspicious for meningitis or ventriculitis [15]. If the patient did not exhibit clinical symptoms or had normal levels of glucose, protein, and nucleated cells, the positive CSF culture was regarded as contaminated. The clinical outcomes were defined as follows: (i) *the 30-day mortality*: death within 30 days after the first CSF bacterial culture was positive; (ii) *CSF sterilization*: a positive CSF culture, followed by at least one and all subsequent CSF cultures being negative after treatment [21].

### Data collection

Data of the patients were extracted from electronic medical records, including demographics, primary disease, comorbidities, craniocerebral surgery, length of hospital stay, intensive care unit (ICU) admission, acute physiology and chronic health evaluation II (APACHE II) score, Glasgow coma scale (GCS) on admission, sequential organ failure assessment (SOFA) score using the worst physiological parameters within 24 h before the CSF culture was positive. The data were also extracted from laboratory test data, including CSF white blood cell count, CSF glucose, CSF protein levels, CSF bacterial culture results, antimicrobial use, and treatment efficacy.

### Microbiology

Drug susceptibility tests were carried out using a VITEK 2 Compact automated microbiological analysis system (bioMérieux, France) or the disk diffusion method according to the Clinical and Laboratory Standards Institute (CLSI) criteria in our microbiology laboratory. The results were interpreted in accordance with the CLSI 2021 criteria, and Enterobacteriaceae with minimum inhibitory concentration (MIC) ≥ 4 mg/L and *A. baumannii* and *P. aeruginosa* with MIC ≥ 8 mg/L were considered resistant to carbapenem [22].

### Statistical analysis

Statistical analysis was performed using SPSS version 25.0. Measurement data were expressed as median and interquartile range (IQR) for continuous variables and percentages (%) for categorical variables. Normally distributed continuous variables were analyzed by the *t* test, while non-normally variables were analyzed by the Mann–Whitney test. The chi-square test and Fisher’s exact test were used to compare categorical variables. The 30-day survival was compared with Kaplan–Meier analysis and log-rank test. Variables with p-values < 0.1 were included in multivariate logistic regression to estimate odds ratios (ORs) for 30-day mortality. All p-values were two-tailed, and a p-value < 0.05 was considered statistically significant.

## Results

### Clinical characteristics

**Fig. 1:**
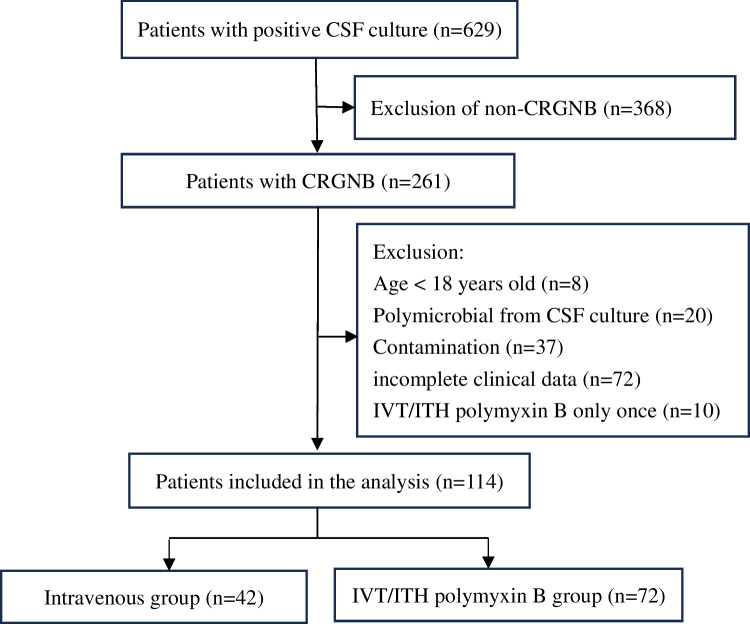
Flowchart of patient enrollment. CSF, cerebrospinal fluid; CRGNB, carbapenem-resistant gram-negative bacteria; IVT/ITH, intraventricular/intrathecal

A total of 629 patients with positive CSF cultures were retrospectively reviewed, of which 515 were excluded, and 114 were finally enrolled. There were 72 patients in the IVT/ITH group, and 42 in the IV group (Fig. 1). The most prevalent reason for admission was cerebrovascular disease (65/114, 57.0%), followed by craniocerebral trauma (34/114, 29.8%) and intracranial tumors (15/114, 13.2%). A comparison of the clinical features of the two patient groups is revealed in Table 1. Compared with the IV group, patients in the IVT/ITH group were older (*p* < 0.001), and fewer patients received intravenous carbapenems (*p* = 0.038). There was a higher CSF sterilization in 7 days (*p* < 0.001), and lower 30-day mortality was observed (*p* = 0.032). In the IVT/ITH group, the median duration of IVT/ITH polymyxin B was 10 days, with a cumulative dose of 45 mg. Among them, one patient experienced tremors after ITH polymyxin B; the symptoms were mild and completely resolved after withdrawal.

**Table 1:** Clinical characteristics and outcomes of patients

Variable	IVT/ITH group (*n* = 72)	IV group (*n* = 42)	*p* value
Age, years	58 [49, 66]	48 [36.5, 55]	< 0.001
Male, *n* (%)	39 (54.2%)	26 (61.9%)	0.421
Primary disease, *n* (%)			
Craniocerebral trauma	21 (29.2%)	13 (31.0%)	0.122
Intracranial tumour	13 (18.1%)	2 (4.8%)	
Cerebrovascular disease	38 (52.8%)	27 (64.3%)	
Comorbidities, *n* (%)			
Hypertension	27 (37.5%)	13 (31.0%)	0.480
Diabetes	11 (15.3%)	1 (2.4%)	0.065
ICU admission, *n* (%)	65 (90.3%)	37 (88.1%)	0.960
SOFA score	4 [2, 5]	4 [3, 6]	0.605
GCS on admission	8 [5, 13]	8 [5, 10]	0.196
APACHE II score	18 [15, 23]	20 [17, 22]	0.921
Any surgeries before infection, *n* (%)			
Craniotomy evacuation of hematoma + decompressive craniectomy	30 (41.7%)	15 (35.7%)	0.531
Lumbar cistern drainage	26 (36.1%)	10 (23.8%)	0.173
External ventricular drainage	19 (26.4%)	17 (40.5%)	0.119
Intracranial tumor resection	13 (18.1%)	2 (4.8%)	0.043
Craniotomy aneurysm clipping	12 (16.7%)	7 (16.7%)	1.000
Aneurysm embolization	7 (9.7%)	0 (0.0%)	0.093
Ventricle peritoneal shunt	3 (4.2%)	5 (11.9%)	0.238
Initial fever, mean, ℃	38.8 ± 0.8	38.7 ± 0.8	0.364
Initial CSF WBC, cells/µL	3500 [1014,11150]	1886 [574, 4970]	0.066
Initial CSF Glucose, mmol/L	0.07 [0.02, 0.56]	0.49 [0.03, 1.31]	0.060
Initial CSF Protein, mg/dL	289.8 [180.1, 353.15]	248.6 [172.3, 356.9]	0.533
Gram-negative bacteria in CSF, n (%)			
* Acinetobacter baumannii*	45 (62.5%)	27 (64.3%)	0.867
* Klebsiella pneumoniae*	24 (33.3%)	12 (28.6%)	
* Pseudomonas aeruginosa*	2 (2.8%)	2 (4.8%)	
Other CRE	1 (1.4%)	1 (2.4%)	
Bloodstream infection	11 (15.3%)	8 (19.0%)	0.602
AKI	6 (8.3%)	1 (2.4%)	0.383
Surgical treatment after infection ^a^, *n* (%)	61 (84.7%)	32 (76.2%)	0.257
Intravenous carbapenems	39 (54.2%)	31 (73.8%)	0.038
IVT/ITH administration of polymyxin B			
Duration, days	10 [5, 17]		
Cumulative dose, mg	45 [25,60]		
ICU length of stay, days	27 [13,51]	17 [6, 46]	0.228
Hospital length of stay, days	42 [28,58]	34 [20, 59]	0.172
CSF sterilisation in 7 days, *n* (%)	46 (63.9%)	11 (26.2%)	< 0.001
30-day mortality	20 (27.8%)	20 (47.6%)	0.032

*IVT *intraventricular, *ITH *intrathecal, *IV* intravenous, *ICU *intensive care unit, *SOFA *sequential organ failure assessment, *GCS *Glasgow coma scale, *APACHE II* acute physiology and chronic health evaluation II, *CSF *cerebrospinal fluid, *WBC *white blood cell, *CRE *carbapenem-resistant Enterobacteriaceae, *AKI *Acute Kidney Injury

^a^ including removal, replacement, or insertion of new intracranial devices, and debridement

### Microbiology

All CSF cultured demonstrated CRGNB, including 72 cases (63.2%) of carbapenem-resistant *A. baumannii* (CRAB) infection, 36 (31.6%) of carbapenem-resistant *K. pneumoniae* (CRKP) infection, 4 (3.5%) of carbapenem-resistant *P. aeruginosa* (CRPA) infection, and 2 (1.8%) of other carbapenem-resistant Enterobacteriaceae (CRE) infection (Table 1). All *A. baumannii* and *K. pneumoniae* isolates were resistant to carbapenems and were susceptible to polymyxin B (Table 2).

**Table 2:** Antibiotic resistance of carbapenem-resistant gram-negative bacteria (CRGNB) isolates in cerebrospinal fluid (CSF)

Antibiotics	*Acinetobacter baumannii* (*n* = 72)	*Klebsiella pneumoniae* (*n* = 36)	*Pseudomonas aeruginosa* (*n* = 4)	Other CRE (*n* = 2)
Ceftazidime	97.2 (69/71)	100.0(35/35)	50.0 (2/4)	100.0 (2/2)
Cefepime	94.4 (68/72)	83.3 (30/36)	75.0 (3/4)	0.0 (0/2)
Carbapenem	100.0 (72/72)	100.0(36/36)	100.0 (4/4)	100.0 (2/2)
Cefoperazone/Sulbactam	64.3 (45/70)	100.0(36/36)	100.0 (4/4)	100.0 (2/2)
Levofloxacin	62.5 (45/72)	83.3 (30/36)	75.0 (3/4)	0.0 (0/2)
Tigecycline	3.1 (2/64)	13.3 (4/30)	-	0.0 (0/1)
Amikacin	35.4 (23/65)	41.7 (15/36)	0.0 (0/4)	0.0 (0/2)
Polymyxin B	0.0 (0/60)	0.0 (0/27)	0.0 (0/4)	0.0 (0/1)

*CRE *carbapenem-resistant Enterobacteriaceae

### CRKP and CRAB infections

**Fig. 2:**
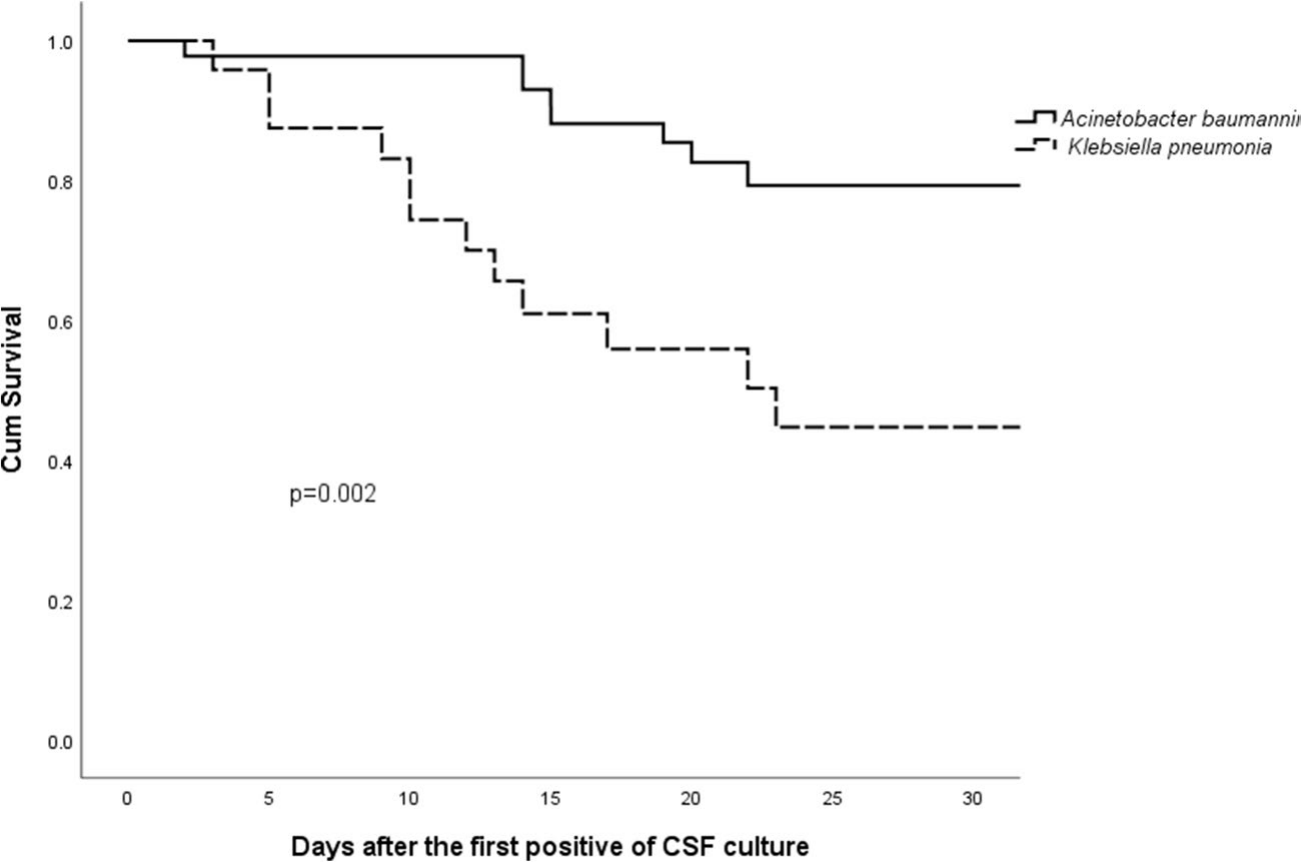
Kaplan-Meier curves of 30-day mortality after the first CSF bacteria culture positive in IVT/ITH group

In the IVT/ITH group, patients with CRKP infection tended to have a higher initial fever (*p* = 0.014), higher incidence of bloodstream infection (*p* = 0.040), and lower CSF sterilization in 7 days (*p* < 0.001) than those with CRAB infection. In addition, a significant difference was observed in males (*p* = 0.036), combined intravenous carbapenems (*p* = 0.015), and IVT/ITH administration (*p* = 0.035) between the two groups. Patients with CRKP infection had a higher 30-day mortality rate than those with CRAB infection (50.0% vs. 17.8%, *p* = 0.005; Table 3). Kaplan–Meier curves also demonstrated a significant difference between the two groups (*p* = 0.002; Fig. 2). However, no significant difference was found between CRAB and CRKP infections in the IV group (Table 3).

**Table 3:** Clinical characteristics and outcomes of patients with CRAB and CRKP infection

	IVT/ITH group			IV group		
Variable	CRAB (*n* = 45)	CRKP (*n* = 24)	*p* value	CRAB (*n* = 27)	CRKP (*n* = 12)	*p* value
Age, years	58 [52, 65]	59 [46, 67]	0.982	48 [37, 55]	51 [36, 59]	0.778
Male, *n*%	20 (44.4%)	17 (70.8%)	0.036	14 (51.9%)	9 (75.0%)	0.291
Primary disease, *n*%						
Craniocerebral trauma	13 (28.9%)	6 (25.0%)	0.476	7 (25.9%)	4 (33.3%)	0.802
Intracranial tumour	6 (13.3%)	6 (25.0%)		1 (3.7%)	0 (0.0%)	
Cerebrovascular disease	26 (57.8%)	12 (50.0%)		19 (70.4%)	8 (66.7%)	
Comorbidities, *n*%						
Hypertension	19 (42.2%)	8 (33.3%)	0.471	10 (37.0%)	3 (25.0%)	0.714
Diabetes	8 (17.8%)	3 (12.5%)	0.736	1 (3.7%)	0 (0.0%)	1.000
ICU admission, *n*%	43 (95.6%)	21 (87.5%)	0.333	25 (92.6%)	11 (91.7%)	1.000
SOFA score	4 [2, 5]	4 [3, 5]	0.861	5 [3, 6]	4 [3, 6]	0.944
GCS on admission	7 [5, 11]	8.5 [5, 14.5]	0.419	8 [5, 9]	7 [4, 9]	0.593
APACHE II score	18.5 [15, 22]	18 [14, 24]	0.641	20 [17, 21]	19 [16, 23]	0.681
Initial fever, mean, ℃	38.7 ± 0.8	39.2 ± 0.6	0.014	38.5 ± 0.8	38.6 ± 0.6	0.994
Initial CSF WBC, cells/µL	3200 [1225,8460]	5180 [1010.5,23840]	0.153	2080 [520, 6880]	2251 [677, 5400]	0.975
Initial CSF Glucose, mmol/L	0.065 [0.02,0.30]	0.11 [0.015,1.00]	0.586	0.55 [0.03, 1.70]	0.05 [0.01, 0.68]	0.179
Initial CSF Protein, mg/dL	278.65 [176.80,340.67]	322.45 [180.45,369.32]	0.215	278.55 [165.13, 361.35]	280.90 [191.80, 437.00]	0.574
Bloodstream infection, *n* (%)	4 (8.9%)	7 (29.2%)	0.040	5 (18.5%)	3 (25.0%)	0.682
AKI, n (%)	5 (11.1%)	1 (4.2%)	0.657	1 (3.7%)	0 (0.0%)	1.000
Surgical treatment after infection ^a^, *n* (%)	39 (86.7%)	19 (79.2%)	0.496	19 (70.4%)	8 (29.6%)	0.228
Intravenous carbapenems, *n* (%)	20 (44.4%)	18 (75.0%)	0.015	17 (63.0%)	11 (91.7%)	0.122
IVT/ITH administration of polymyxin B						
IVT injection, *n* (%)	9 (20.0%)	9 (37.5%)	0.035			
ITH injection, *n* (%)	34 (75.6%)	11 (45.8%)				
IVT + ITH injection, *n* (%)	2 (4.4%)	4 (16.7%)				
Duration, days	10 [5.5, 16]	9.5 [6, 19]	0.545			
Cumulative dose, mg	45 [25,60]	40 [26.25,65]	0.432			
IVT/ITH administration within 48 h after the CSF culture results, n (%)	23 (51.1%)	10 (41.7%)	0.454			
ICU length of stay, days	27 [13,52.5]	23 [11,49.5]	0.203	18 [6, 45]	21 [10, 71]	0.353
Hospital length of stay, days	43 [28,63.5]	36.5 [24, 55]	0.132	32 [15, 60]	38 [27, 71]	0.344
CSF sterilisation in 7 days, *n* (%)	37 (82.2%)	6 (25.0%)	< 0.001	6 (22.2%)	4 (33.3%)	0.693
30-day mortality, *n* (%)	8 (17.8%)	12 (50%)	0.005	15 (55.6%)	5 (41.7%)	0.423

*IVT *intraventricular, *ITH *intrathecal, *IV* intravenous, *CRAB *carbapenem-resistant *Acinetobacter baumannii*, *CRKP *carbapenem-resistant *Klebsiella pneumoniae*, *ICU* intensive care unit, *SOFA *sequential organ failure assessment, *GCS *Glasgow coma scale, *APACHE II* acute physiology and chronic health evaluation II, *CSF *cerebrospinal fluid, *WBC* white blood cell, *AKI *Acute Kidney Injury

^a^ including removal, replacement, or insertion of new intracranial devices, and debridement

### Factors related to 30-day all-cause mortality in patients with IVT/ITH polymyxin B

The 30-day all-cause mortality rate in patients treated with IVT/ITH polymyxin B was 27.8% (20/72). Univariate analysis depicted that CRKP infection (*p* = 0.003), bloodstream infection (*p* = 0.031), IVT or ITH administration (*p* = 0.027), duration of IVT/ITH polymyxin B (*p* = 0.008), combined intravenous carbapenems (*p* = 0.028), and CSF sterilization in 7 days (*p* = 0.009) were associated with 30-day mortality. Multivariate logistic regression analysis revealed that the duration of IVT/ITH polymyxin B (*p* = 0.021) was an independent risk factor associated with 30-day mortality (Table 4).

**Table 4:** Univariate and multivariate logistic regression analysis for 30-day mortality in patients with IVT/ITH administration

Variable	Survivors(*n* = 52)	Non-survivors(*n* = 20)	*p* value	OR (95% CI)	*p* value
Initial fever, mean, ℃	38.7 ± 0.8	39.1 ± 0.7	0.064		
CRKP infection, *n* (%)	12 (23.1%)	12 (60.0%)	0.003		
Bloodstream infection, *n* (%)	5 (9.6%)	6 (30.0%)	0.031		
IVT or ITH administration					
IVT injection, *n* (%)	11 (21.2%)	9 (45.0%)	0.027		
ITH injection, *n* (%)	38 (73.1%)	8 (40.0%)			
IVT + ITH injection, *n* (%)	3 (5.8%)	3 (15.0%)			
Duration of IVT/ITH polymyxin B, days	11 [6, 20]	7.5 [5, 10]	0.008	0.87 (0.77–0.98)	0.021
Combined intravenous carbapenems, *n* (%)	24 (46.2%)	15 (75.0%)	0.028		
CSF sterilisation in 7 days, *n* (%)	38 (73.1%)	8 (40.0%)	0.009		

*CRKP *carbapenem-resistant *Klebsiella pneumoniae*, *IVT* intraventricular, *ITH* intrathecal, *CSF* cerebrospinal fluid

## Discussion

This study retrospectively evaluated patients with post-neurosurgical intracranial infection caused by CRGNB and observed that patients receiving IVT/ITH polymyxin B had increased CSF microbiological eradication and reduced mortality. In addition, for patients receiving IVT/ITH administration, the mortality of patients with CRKP infection was significantly higher than that of patients with CRAB infection.

CRGNB is associated with increased mortality [23]. CRAB has become the most common pathogen for nosocomial intracranial infection neurosurgery wards [5, 24]. A retrospective study showed that the 30-day mortality of postoperative CNS infection caused by MDR/XDR *A. baumannii* was 28.6%, even after IVT/ITH administration [25]. Notably, the pathogenicity and toxicity of CRKP are higher than those of CRAB, resulting in high mortality [26]. A retrospective study displayed that ICU admission, bacteremia, and hospital-acquired pneumonia were risk factors for CRE meningitis, and the overall mortality of CRE meningitis/encephalitis was as high as 69.2% [27]. To date, few studies or cases have reported IVT/ITH administration in treating intracranial infections caused by CRKP, most of which were retrospective studies [28–31]. In our study, the mortality of patients with intracranial infections caused by CRKP remained high even with IVT/ITH polymyxin B, but we also found that patients with CRKP infection had more combined bloodstream infections than those with CRAB infection. Mortality in patients with CRGNB infection was linked to various factors, including age, SOFA score, Charlson Index and bacteremia [23, 32]. Consequently, large-scale clinical studies of CRKP intracranial infection are still needed.

Although the preferred approach for the treatment of CRGNB intracranial infections remains unclear, a combination of intravenous and IVT/ITH therapy may be necessary for optimal clinical outcomes [33]. Combining IVT with intravenous administration is likely to achieve higher antibiotic levels in CSF and more effective sterilization than IVT therapy alone [21, 34], This combination may help prevent compartments with subinhibitory antibiotic concentrations, thereby reducing the probability of the selection of resistant bacteria and relapse [35]. In a setting of high prevalence of nosocomial infections caused by CRGNB, polymyxins should be considered in combination with other drugs. Several in vitro and clinical studies have suggested the clinical benefits of adopting polymyxin–drug combination therapy, especially polymyxin plus meropenem, with synergistic killing against MDR/XDR gram-negative bacteria [36–39]. Although meropenem is recommended as the main empirical treatment for healthcare-associated ventriculitis and meningitis against gram-negative bacteria [40], resistance may lead to delayed treatment effectiveness and adverse outcomes. This study found no beneficial results for the combination of polymyxin B and carbapenems, even when carbapenem therapy was optimized, such as increasing the dose and extending the infusion. This indicates that optimizing the combination treatment regimen for intracranial infection caused by CRGNB is extremely challenging.

The current guideline suggests CSF antimicrobial concentrations of 10–20 times the MIC of the isolate and a daily IVT dose of 5 mg polymyxin B for gram-negative ventriculitis and meningitis [15]. The CSF antimicrobial concentrations are affected by the ventricular size and daily drain output, and drain clamping should be performed after each administration (15–60 min). IVT administration ensures distribution throughout CSF compartment, whereas ITH dosing often fails to attain adequate antibiotic concentrations in the ventricles [11]. However, this study did not find significant differences in clinical outcomes between these two regimens.

Prompt diagnosis and intervention using appropriate antibiotics are considered imperative. A case series and systematic review demonstrated that death was related to delayed ITH/IVT therapy compared with survival (7 vs. 2 days, *p* = 0.01) [41]. Furthermore, studies on the association between the timing of ITH/IVT administration and clinical outcomes are sparse. The results of this study showed that timely IVT/ITH treatment within 48 h of a positive CSF culture had no significant effect on clinical outcome.

The length of IVT delivery varied among the available studies, while the total treatment duration has often not been specified [13]. The current guideline recommends that adequate antimicrobial therapy should continue for 21 days by gram-negative bacilli and up to 10–14 days after the last positive culture in patients with repeatedly positive CSF cultures [15]. The median/mean duration of treatment with IVT/ITH polymyxins was 13–15 days in the current small retrospective study [18, 42, 43], which is similar to the results of this study.

In a recent meta-analysis, the incidence of complications in 229 patients with meningitis caused by gram-negative pathogens who received intrathecal administration was as high as 13%, and chemical meningitis and seizures represented the majority of the complications [44]. However, the adverse effects of IVT/ITH administration reported in previous studies were mainly due to colistin rather than polymyxin B [20]. Since patients receiving IVT/ITH therapy are usually in a coma or are sedated, adverse drug effects may have gone unnoticed or been attributed to complications of the underlying disease, and the true incidence of adverse effects of IVT/ITH therapy may be underestimated.

There are some limitations to the current study. First, this was a retrospective study with a small sample size in a single center and relied on clinical culture results, which may introduce bias in data interpretation. Second, as patients were seriously ill and other confounding factors were common in the ICU, we could not identify the impact of these factors on mortality. Third, the modified Rankin scale or Glasgow Outcome Scale were not used, as some patients may survive and present in a vegetative state, which is also a devastating outcome. Finally, the effect of ventricular size and daily CSF drainage volume on the outcome of IVT/ITH administration was not analyzed, and further multicenter randomized controlled studies are required.

## Conclusions

Intravenous combined with IVT/ITH polymyxin B increased CSF microbiological eradication and reduced mortality. CRKP intracranial infection may lead to more difficult treatment and worse outcome, requiring attention and further optimized treatment.

## Data Availability

The datasets generated during and/or analyzed during the current study are not publicly available due to institutional ethics, privacy, and confidentiality regulations, but are available from the corresponding author on reasonable request.
